# High Avidity dsDNA Autoantibodies in Brazilian Women with Systemic Lupus Erythematosus: Correlation with Active Disease and Renal Dysfunction

**DOI:** 10.1155/2015/814748

**Published:** 2015-10-25

**Authors:** Rodrigo C. Oliveira, Isabela S. Oliveira, Mittermayer B. Santiago, Maria L. B. Sousa Atta, Ajax M. Atta

**Affiliations:** ^1^Programa de Pós-Graduação em Imunologia, Instituto de Ciências da Saúde, Universidade Federal da Bahia, 40140-100 Salvador, BA, Brazil; ^2^Escola Bahiana de Medicina e Saúde Pública, 40050-420 Salvador, BA, Brazil; ^3^Laboratório de Pesquisa em Imunologia, Departamento de Análises Clínicas e Toxicológicas, Faculdade de Farmácia, Universidade Federal da Bahia, Rua Barão de Jeremoabo 147, 40170-115 Salvador, BA, Brazil

## Abstract

We investigated in Brazilian women with SLE the prevalence and levels of high avidity (HA) dsDNA antibodies and tested their correlation with lupus activity and biomarkers of renal disease. We also compared these correlations to those observed with total dsDNA antibodies and antibodies against nucleosome (ANuA). Autoantibodies were detected by ELISA, while C3 and C4 levels were determined by nephelometry. Urine protein/creatinine ratio was determined, and lupus activity was measured by SLEDAI-2K. The prevalence of total and HA dsDNA antibodies was similar to but lower than that verified for ANuA. The levels of the three types of antibodies were correlated, but the correlation was more significant between HA dsDNA antibodies and ANuA. High avidity dsDNA antibodies correlated positively with ESR and SLEDAI and inversely with C3 and C4. Similar correlations were observed for ANuA levels, whereas total dsDNA antibodies only correlated with SLEDAI and C3. The levels of HA dsDNA antibodies were higher in patients with proteinuria, but their levels of total dsDNA antibodies and ANuA were unaltered. High avidity dsDNA antibodies can be found in high prevalence in Brazilian women with SLE and are important biomarkers of active disease and kidney dysfunction.

## 1. Introduction

In lupus, there is an important autoreactivity of B lymphocytes shown by the production of more than 160 specificities of autoantibodies and circulating immune complexes of autoantibodies and autoantigens [1–3]. The dsDNA autoantibody is the most important laboratory biomarker of SLE associated with both disease activity and renal dysfunction. However, the autoantibody's involvement in lupus immunopathogenesis still deserves more investigation [4–6]. Although this antibody shows high SLE specificity, its prevalence in different studies has been estimated to be around 50% [7, 8]. In addition, dsDNA antibodies can be found in patients regardless of whether they have renal disease. Interestingly, dsDNA antibodies exhibit a high degree of heterogeneity, as shown by their cross-reactions with other autoantigens and different isotypes as well as by changes in their affinity to bind dsDNA epitopes [9–11]. This study investigated the prevalence of dsDNA autoantibodies of high avidity and their correlations with clinical and laboratory findings in SLE patients living in northeastern Brazil. In addition, these correlations were compared to those obtained with total dsDNA antibodies and nucleosome antibodies.

## 2. Material and Methods

### 2.1. Patients

One hundred forty-two SLE female patients from the Rheumatology Service of the Santa Izabel Hospital (Salvador, Bahia) were consecutively enrolled in this study. All had a previous diagnosis of lupus and exhibited four or more criteria for SLE [12]. Lupus activity was scored with the SLEDAI-2K [13]. Prednisone was the main medication used by the patients (132/142, 93.0%), combined with Chloroquine or Chloroquine plus Azathioprine (89/132, 67.4%). Thirty-two patients (32/132, 24.2%) were taking Methotrexate plus Prednisone and Chloroquine, combined or not with Azathioprine. Cyclosporine was rarely used (7/132, 5.3%). All patients signed an informed consent form to participate in this study, which was approved by the Ethics Committee of the Santa Izabel Hospital.

### 2.2. Laboratory Investigation

Anti-dsDNA IgG antibodies were first tested by the indirect fluorescent antibody test with* Crithidia luciliae* (CLIFT), followed by an indirect ELISA to measure their serum levels (Orgentec Diagnostika GmbH, Germany). Afterward, the presence and levels of high avidity dsDNA IgG antibodies were measured with the QUANTA Lite test HA dsDNA ELISA (INOVA Diagnostics Inc., San Diego, CA, USA). The cutoffs in the ELISA test were 25 IU/mL and 30 IU/mL, respectively. An indirect ELISA, using a cutoff of 20 U/mL (Orgentec), determined the levels of nucleosome antibodies. Cellular analysis of blood was done with the cytometer CellDyn-Ruby (Abbot Diagnostic Inc., USA) while inflammation was measured by erythrocyte sedimentation rate. Serum levels of complement C3 (reference range = 67–149 mg/dL) and C4 (reference range = 10–38 mg/dL) were determined by nephelometry in the Image Immunochemistry System (Beckman-Coulter, USA). In addition, the presence of renal dysfunction was obtained by chemical and microscopic examination of fresh urine using the analyzer LabUMat UriSed (Electronic Muszeripari Kft, Budapest). Colorimetric methods measured the levels of urine protein and urine creatinine. In this study, a significant proteinuria was a urine protein/creatinine ratio (P/C ratio) >0.23. This cutoff was calculated with a receiver operating characteristics (ROC) curve using the P/C ratios of patients having negative or positive diagnosis of lupus nephritis when they were included in the study (cutoff = 0.23, AUC = 0.904; sensitivity = 88.5%, specificity = 80.3%).

### 2.3. Statistical Analysis

The test of D'Agostino and Pearson analyzed the distribution of the continuous variables, which were presented as mean ± SD or median and interquartile range (IQR, 25−75%). The test of Spearman performed correlation analyses, which were validated for their statistical significance in accordance with the number of* XY* pairs tested. The means and medians of two groups were compared with the unpaired *t*-test and *U* test of Mann-Whitney, respectively. The significance level was significant at *P* < 0.050. The statistical software GraphPad 6.0 and MedCalc 13.0 were used.

## 3. Results

### 3.1. Clinical and Demographic Data

Lupus patients had a mean age of 40.6 ± 12.9 years (95% CI = 38.5–42.7 years), ranging from 17 to 79 years. The median of lupus duration in these women was eight years, varying from 0.4 to 40 years. One hundred twenty-five patients (88.0%) had active lupus. In 80/142 (56.3%) patients, the activity varied from moderate to very high (median = 9, range 6–31). Sixty patients had a clinical diagnosis of kidney disorder demonstrated by proteinuria and presence of urine leukocytes, erythrocytes, and less frequently urinary casts [14]. Autoantibodies and low C3 and C4 levels were found in different prevalence in the patients, predominating ANA, AnuA, and dsDNA antibodies (Table 1).

### 3.2. Correlation Analysis

There was a correlation between the levels of total dsDNA antibodies and HA dsDNA antibodies (*XY* pairs = 66, *r* = 0.50; *P* < 0.0001). On the other hand, the levels of total dsDNA antibodies and of HA dsDNA antibodies were correlated with ANuA levels (*XY* pairs = 70, *r* = 0.34; *P* = 0.004 and* XY* pairs = 65, *r* = 0.61, *P* < 0.0001, resp.). However, the correlation between HA dsDNA antibodies and nucleosome antibodies was higher (*P* = 0.044). The levels of total dsDNA antibodies were only correlated with SLEDAI scores and C3 levels. Differently, the levels of both HA dsDNA antibodies and ANuA, besides correlating with SLEDAI scores and the C3 levels, were also correlated with C4 levels and ESR (Table 2).

### 3.3. Proteinuria, C3, and Autoantibody Levels

**Figure 1:**
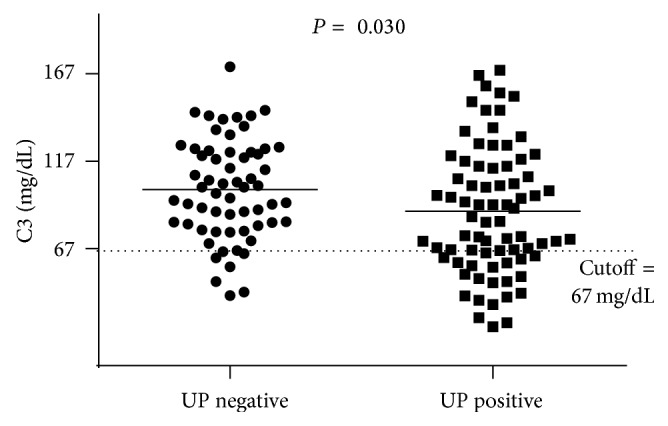
Serum levels of C3 in SLE patients without proteinuria (UP negative, P/C ratio ≤ 0.23) and presenting urine protein (UP positive, P/C ratio > 0.23).

**Figure 2:**
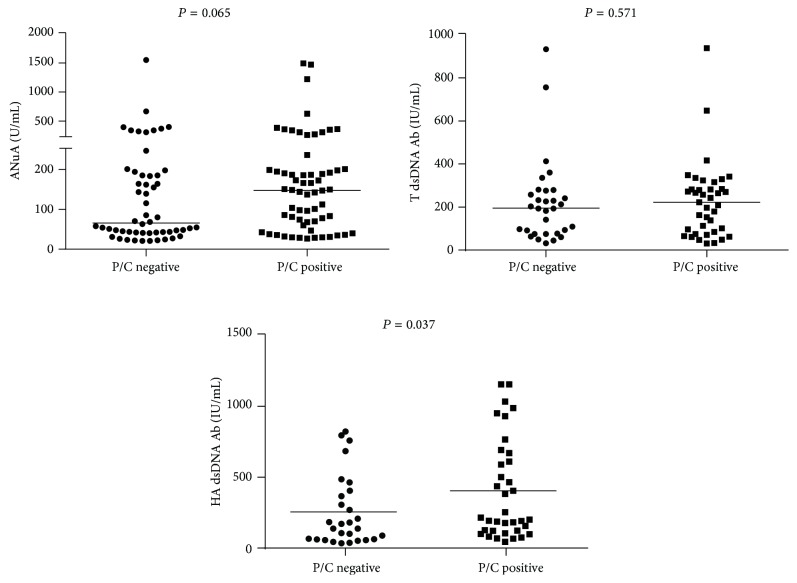
Levels of total dsDNA antibodies, HA dsDNA antibodies, and ANuA in SLE Brazilian women without and with proteinuria (P/C ratio ≤ 0.23 and >0.23, resp.). The medians are represented by horizontal lines and were compared with the *U* test of Mann-Whitney.

The serum levels of C3 were lower in the patients with a P/C ratio >0.23 (Figure 1). There was a difference between the levels of HA dsDNA antibodies in patients with and without proteinuria (P/C ratio > 0.23; *P* = 0.037). However, the levels of total dsDNA antibodies and ANuA were similar in these two groups of patients (*P* = 0.571 and *P* = 0.065, resp.) (Figure 2).

## 4. Discussion

Anti-dsDNA IgG autoantibodies are important biomarkers in systemic lupus erythematosus. Nevertheless, the Farr RIA or another immunoassay to detect high avidity dsDNA antibodies is not routinely used in the rheumatology laboratory, being widely substituted by ELISA tests that measure total dsDNA antibody levels. These immunoassays do not discriminate between antibodies of low and high affinity or antibodies that cross-react with dsDNA epitopes. In contrast, ELISA tests that detect HA dsDNA are comparable to Farr RIA [10, 15–19]. Although dsDNA antibodies have been associated with lupus activity and lupus nephritis, the role of these antibodies in SLE pathogenesis still deserves more study. To date, renal disease has been demonstrated in a large proportion of SLE patients who are seronegative for dsDNA antibodies and can be absent in patients who have high levels of these autoantibodies.

Previously, we did not find an association between laboratory findings of lupus kidney disease such as proteinuria and altered urine exam in Brazilian patients with high levels of total dsDNA antibodies. Such observation suggested the need of more studies to characterize the avidity of these autoantibodies [20]. In the present study, nucleosome antibodies were correlated with total dsDNA antibodies and more strongly with HA dsDNA antibodies. This finding was expected because nucleosome is a molecular complex constituted by histones, nonhistone proteins, and dsDNA. Thus, the presence of dsDNA epitopes in nucleosome can elicit specific autoantibodies that also participate in the immune reactions of tests that detect dsDNA antibodies, mainly of high avidity, justifying these correlations.

Herein, we demonstrated that total dsDNA antibodies measured by a routine indirect ELISA can present a correlation with lupus activity and C3 levels. However, the levels of HA dsDNA antibodies and ANuA, besides exhibiting good correlation with SLEDAI and C3 levels, were also correlated with low C4 levels and ESR. In lupus, immune complexes formed by IgG and IgM autoantibodies and self-antigens activate complement lowering both C3 and C4 levels. Both low C3 and C4 are biomarkers of disease activity and were recently included as immunologic criteria for SLE by the Systemic Lupus International Collaborating Clinics (SLICC) group [21]. Together with dsDNA antibodies, low C3 and C4 are also biomarkers of lupus nephritis, but low C3 levels seem to be more sensitive than low C4 levels to diagnose renal SLE flares. In the present work, C3 levels were more strongly correlated with the levels of HA dsDNA antibodies, being that this correlation was higher than that of C4 levels. In contrast with C4 levels, C3 levels were lower in the patients with renal disorder. Interestingly, only the levels of HA dsDNA antibodies were higher in SLE patients with proteinuria, here demonstrated by a urine P/C ratio above 0.23. Compared with 24 h urine protein, the use of spot urine P/C ratio still is controversial. However, several studies have supported the use of P/C ratio in the clinical practice, and it has been recently adopted by the SLICC study [21]. The findings presented here do not exclude the participation of other immune mediators in the pathogenesis of kidney disease in these individuals. Thus, the contribution of C1q antibodies, as well as the involvement of different isotypes of dsDNA antibodies, activated T lymphocytes, or inflammatory cytokines, must also be considered [22–24].

In conclusion, HA dsDNA antibodies can be found with high prevalence in Brazilian women with SLE and seem to be important biomarkers of active disease and contribute to kidney dysfunction in these patients.

## Figures and Tables

**Table 1:** Immunological findings in Brazilian SLE women.

Immune marker	Prevalence *N* 142 (%)	Level (median, IQR)
ANA	131 (92.2)	320 (160–1,280)
ANuA (U/mL)	118 (83.1)	114 (44–196)
Total dsDNA Ab (IU/mL)	72 (50.7)	205 (78–279)
HA dsDNA Ab (IU/mL)	66 (46.5)	189 (95–525)
Sm Ab (U/mL)	35 (24.6)	72 (51–335)
RNP-70 Ab (U/mL)	48 (33.8)	106 (91–244)
SS-A/Ro Ab (U/mL)	55 (38.7)	87 (56–221)
SS-B/La Ab (U/mL)	13 (9.1)	101 (45–304)
Rib-P Ab (U/mL)	20 (14.1)	18 (16–190)
IgA anti-*Β*2GPI (U/mL)	32 (22.5)	26 (19–41)
IgG anti-*Β*2GPI (U/mL)	16 (11.3)	20 (12–34)
IgM anti-*Β*2GPI (U/mL)	9 (6.3)	22 (19–53)
IgA aCL (U/mL)	3 (<5.0)	38 (15–40)
IgG aCL (U/mL)	11 (7.7)	18 (10–37)
IgM aCL (U/mL)	7 (<5.0)	36 (26–65)
C3 low (<67 mg/mL)	36 (25.3)	57 (41–64)
C4 low (<10 mg/mL)	29 (20.4)	5 (4–8)

**Table 2:** Correlation of total dsDNA antibodies, HA dsDNA antibodies, and ANuA with clinical and laboratory findings in SLE patients.

	Total dsDNA Ab	HA dsDNA Ab	ANuA
SLEDAI	0.33 0.005	0.45 0.0001	0.43 <0.0001

ESR	0.29 NS	0.36 0.003	0.37 <0.0001

C3	−0.34 0.003	−0.55 <0.0001	−0.32 <0.001

C4	−0.24 NS	−0.34 0.006	−0.28 0.002

P/C ratio	0.12 NS	0.24 NS	0.24 NS

Total dsDNA antibody, *XY* pairs = 72; HA dsDNA antibody, *XY* pairs = 66; ANuA, *XY* pairs = 118. NS: not significant.
